# *Helicobacter pylori*-induced premature senescence of extragastric cells may contribute to chronic skin diseases

**DOI:** 10.1007/s10522-017-9676-x

**Published:** 2017-01-10

**Authors:** Anna Lewinska, Maciej Wnuk

**Affiliations:** Department of Genetics, University of Rzeszow, Werynia 502, 36-100 Kolbuszowa, Poland

**Keywords:** *Helicobacter pylori*, Chronic skin diseases, Senescence, Inflammation

## Abstract

*Helicobacter pylori,* one of the most frequently observed bacterium in the human intestinal flora, has been widely studied since Marshall and Warren documented a link between the presence of *H. pylori* in the gastrointestinal tract and gastritis and gastric ulcers. Interestingly, *H. pylori* has also been found in several other epithelial tissues, including the eyes, ears, nose and skin that may have direct or indirect effects on host physiology and may contribute to extragastric diseases, e.g. chronic skin diseases. More recently, it has been shown that *H. pylori* cytotoxin CagA expression induces cellular senescence of human gastric nonpolarized epithelial cells that may lead to gastrointestinal disorders and systemic inflammation. Here, we hypothesize that also chronic skin diseases may be promoted by stress-induced premature senescence (SIPS) of skin cells, namely fibroblasts and keratinocytes, stimulated with *H. pylori* cytotoxins. Future studies involving cell culture models and clinical specimens are needed to verify the involvement of *H. pylori* in SIPS-based chronic skin diseases.

## What are the causes of chronic skin diseases?

Despite the fact that chronic skin diseases, such as erythema, psoriasis, Henoch-Schönlein purpura, alopecia areata, Sweet disease, chronic urticaria, systemic sclerosis, Behcet disease, generalized pruritus (itch), nodular prurigo, lichen planus, aphthous ulceration, Sjögren syndrome and atopic dermatitis, are frequently diagnosed and comprehensively studied, they are still considered as troublesome human diseases of a complex etiopathogenesis (Hernando-Harder et al. 2009; Mogaddam et al. 2015; Wedi and Kapp 2002). It is speculated that approximately ten percent of worldwide population is affected by atopic dermatitis (AD) (Weidinger and Novak 2016). The causes of AD remain elusive. AD is considered a genetic-based immunological disorder associated with the hypersensitivity of the immune system and aberrant response to antigens (allergens). The questions on hereditary patterns, haplotypes and allergens involved in the pathogenesis of AD are left unanswered (Bieber 2010; Brown 2016; Wuthrich et al. 2007). Thus, there is a need for alternative hypotheses on the mechanisms underlying the initiation and progression of chronic skin diseases/conditions. It seems reasonable to correlate inflammatory skin disorders with stress-induced premature senescence (SIPS) in human skin cells (Bellei et al. 2012) that is also accompanied by senescence-associated secretory phenotype (SASP) (Demaria et al. 2015; Ovadya and Krizhanovsky 2014; Tchkonia et al. 2013) that is primarily a DNA damage response (Rodier et al. 2009). Cellular senescence is a natural mechanism to prevent oncogenic transformation of DNA-damaged somatic cells that is based on permanent inhibition of cell proliferation and cell cycle arrest (Campisi 2011). However, senescent cells are metabolically active or even hyperactive and are able to produce pro-inflammatory factors, namely interleukins, chemokines and growth factors that may have adverse effects on surrounding cells and tissues (Demaria et al. 2015; Kennedy et al. 2014). There are several stress stimuli that can promote/potentiate SIPS, like chemicals (e.g. hydrogen peroxide) (Chen et al. 1998), drugs (e.g. doxorubicin) (Bielak-Zmijewska et al. 2014), nutraceuticals (e.g. curcumin) (Grabowska et al. 2015), nanoparticles (Mytych et al. 2015) as well as bacterial toxins (e.g. pyocyanin or lipopolysaccharide) (Kim et al. 2016; Muller 2006) and viruses (e.g. papillomavirus) (Ren et al. 2013). In this view, it would be essential to understand the relationship between bacterial infection-mediated cellular senescence and systemic diseases, especially in terms of *H. pylori*-mediated SIPS (Saito et al. 2010), SASP, gastric diseases and perhaps extragastric diseases.

## *H. pylori* infection and systematic diseases

*H. pylori* is a microaerophile, a Gram-negative bacterium (bacillus of helical or curved shape) of approximately 0.5–1 μm × 2.5–5 μm (width × length). Due to the presence of diametrically located flagella, *H. pylori* is able to move and colonize under mucosa. In general, *H. pylori* is found in the stomach, especially in the gastric mucosa and duodenum being responsible for gastroduodenal diseases such as peptic ulcer disease or gastric carcinoma (Marshall and Warren 1984). Undoubtedly, *H. pylori* is one of the most widespread pathogen among humans, especially in the gastrointestinal tract, and human-*H. pylori* co-existence is calculated to be approximately for 60,000 years (Linz et al. 2007; Moodley et al. 2012). According to World Health Organization, it is speculated that approximately a half of the population of developed countries and 80% of the population of developing countries is affected by *H. pylori* infection (Linz et al. 2007). Surprisingly, *H. pylori* is able to tolerate a broad range of oxygen concentrations, especially at liquid culture at high cell density, namely it can grow at microaerophilic conditions (<5%) as well as at aerobic conditions (21%) (Bury-Mone et al. 2006). *H. pylori* can also form biofilms as well as transform from its normal helical bacillary morphology to a coccoid morphology as a survival strategy and expansion (Andersen and Rasmussen 2009; Cammarota et al. 2012; Stark et al. 1999). Unique adaptation features of *H. pylori* are probably responsible for occasional or persistent colonization of other human tissues including skin (Testerman and Morris 2014).

Despite numerous studies on the mechanisms of *H. pylori* transmission, data on *H. pylori* routes of transmission are ambiguous. It is suggested that human is a main disease carrier (reservoir of *H. pylori*) and several transmission routes are considered, namely gastro–oral, oral–oral and fecal–oral routes (Brown 2000; Schwarz et al. 2008). Thus, saliva and faeces may be considered important for *H. pylori* transmission. The PCR analysis on 102 human saliva samples revealed that 66 individuals were affected by *H. pylori* (Wnuk et al. 2010). Of course, it should be further examined if genetic material of *H. pylori* is from live or dead bacteria, but the presence of live bacterial cells in saliva has been also documented by others (Li et al. 1996). Thus, the presence of *H. pylori* in saliva may be important not only for the transmission of chronic infections of the gastrointestinal tract, but also for the propagation of chronic skin diseases in humans. So, one can ask a question if chronic skin diseases are a result of the exposition to saliva and/or faeces containing live or dead forms of *H. pylori* with damaged/injured skin. During such second transmission, *H. pylori* may also colonize host skin tissues. Moreover, dead cells of *H. pylori* may also promote inflammation as a response to bacterial antigens released from dead cells. *H. pylori* produces a plethora of virulence factors, namely enzymes, endotoxins and hemolysins that allows for survival at low pH in the stomach, adhesion to host cells, re-programming of host cell cytophysiology and attenuation of immune responses (Backert et al. 2016). On the other hand, *H. pylori*-based virulence factors are responsible for chronic infections of the gastrointestinal tract, especially for chronic gastritis leading to gastric and duodenal ulcers and gastric MALT (mucosa associated lymphoid tissue) lymphoma as a response to prolonged stimulation of immune system (Testerman and Morris 2014).

A potential role of *H. pylori* infection in several extragastric diseases, namely hematological, cardiovascular, neurological, metabolic, autoimmune and dermatological diseases, has been also proposed (Hernando-Harder et al. 2009; Kutlubay et al. 2014; Magen and Delgado 2014; Testerman and Morris 2014; Wedi and Kapp 2002). An association between *H. pylori* infection and skin diseases such as chronic idiopathic urticaria and rosacea has been suggested (Kutlubay et al. 2014). For example, *H. pylori* (*cagA* + strains) was present in 81% of rosacea patients who also had gastric complaints (Argenziano et al. 2003). Eradication of *H. pylori* infection has been reported to be effective in some patients with chronic autoimmune urticaria, psoriasis, alopecia areata and Henoch-Schönlein purpura (Magen and Delgado 2014). *H. pylori* may be considered as a plausible infectious agent for triggering autoimmunity (Magen and Delgado 2014). Cytotoxins produced by *H. pylori* may activate cross-reactive T cells and stimulate the production of autoantibodies (Magen and Delgado 2014). Moreover, *H. pylori* heat shock proteins (HSP) with sequence similarity to human HSP may play a role in the pathogenesis of autoimmune diseases (Magen and Delgado 2014). However, the role of *H. pylori* in the pathogenesis of some dermatological diseases has been also questioned (Kutlubay et al. 2014; Magen and Delgado 2014). Patients with mild to severe psoriasis were not found to be more susceptible to *H. pylori* infection; however, *H. pylori* affected the clinical severity of psoriasis (Campanati et al. 2015). *H. pylori* eradication was reported to have no discernible effect on chronic spontaneous urticaria (CSU) beyond that of standard CSU therapy (Curth et al. 2015). Thus, more epidemiological and clinical studies are needed to investigate the association between *H. pylori* and inflammatory skin diseases.

## *H. pylori* proteins may be considered as drivers of cellular senescence

*H. pylori* produces many proteins that are highly immunogenic and are directly and/or indirectly responsible for multiple pathogen-host interactions during infection. Some of these proteins like antioxidative enzymes, neutrophil-activating protein (HP-NAP) or other virulence factors, namely proteases, lipases, cholesteryl glucosides, adhesins, iron transporters, *O*-lipopolysaccharide may be helpful during human skin invasion by *H. pylori* as well as responsible for local inflammation (Bumann et al. 2002; Testerman and Morris 2014; Zanotti and Cendron 2014)*. H. pylori* secretome can be grouped into different categories, one of them are products of the cytotoxic-associated genes of pathogenicity island (8 proteins) and other toxins (5 proteins) (Zanotti and Cendron 2014). Two secreted cytotoxins (oncoproteins), namely VacA and CagA are particularly important for *H. pylori*-based pathologies.

Vacuolating cytotoxin A (VacA, 88 kDa protein) inhibits the proliferation of epithelial cells, modifies pathways involved in the cytoskeleton reorganization and induces apoptosis by release of cytochrome *c* from mitochondria. VacA is also able to inhibit the proliferation of T lymphocytes and phagocytosis and antigen presentation to T lymphocytes that in turn results in the attenuation of immune responses. VacA can also modify cell junctions between neighbouring gastric epithelial cells (Gebert et al. 2003; Palframan et al. 2012).

Cytotoxin associated protein A (CagA, 120–145 kDa protein) is encoded by *cagA* gene within the cag pathogenicity island (cag PAI). *CagA* gene is presented within 60% of genomes of *H. pylori* isolated from patients (Hatakeyama and Higashi 2005). Cytotoxin CagA is transported to epithelial cells by one-step transport system T4SS from cytosol of bacterial cell to host cell excluding periplasmic space. CagA interacts with host cellular proteins involved in signaling pathways regulating cell proliferation, motility and polarity that modulates the phenotype of host cells (Tohidpour 2016). CagA may promote loss of polarity and activate aberrant ERK signaling in host cells (Saito et al. 2010). In nonpolarized gastric epithelial cells, CagA-induced ERK activation resulted in oncogenic stress, upregulation of the p21^Waf1/Cip1^ cyclin-dependent kinase inhibitor and induction of senescence (Saito et al. 2010). In contrast, in polarized epithelial cells, CagA-mediated ERK signaling suppressed p21^Waf1/Cip1^ expression by activating a guanine nucleotide exchange factor–H1–RhoA–RhoA-associated kinase–c-Myc pathway and c-Myc-mediated upregulation of miR-17 and miR-20a that stimulated mitogenesis (Saito et al. 2010). Thus, CagA may directly induce cellular senescence in host cells, here gastric cells (Saito et al. 2010), that may be important for the etiopathogenesis of gastric ulcer and perhaps during initiation of chronic skin diseases associated with the induction of secretory phenotype in senescent skin cells.

## Gastric and extragastric diseases associated with *H. pylori* may have a common SIPS-based molecular mechanism

**Fig. 1:**
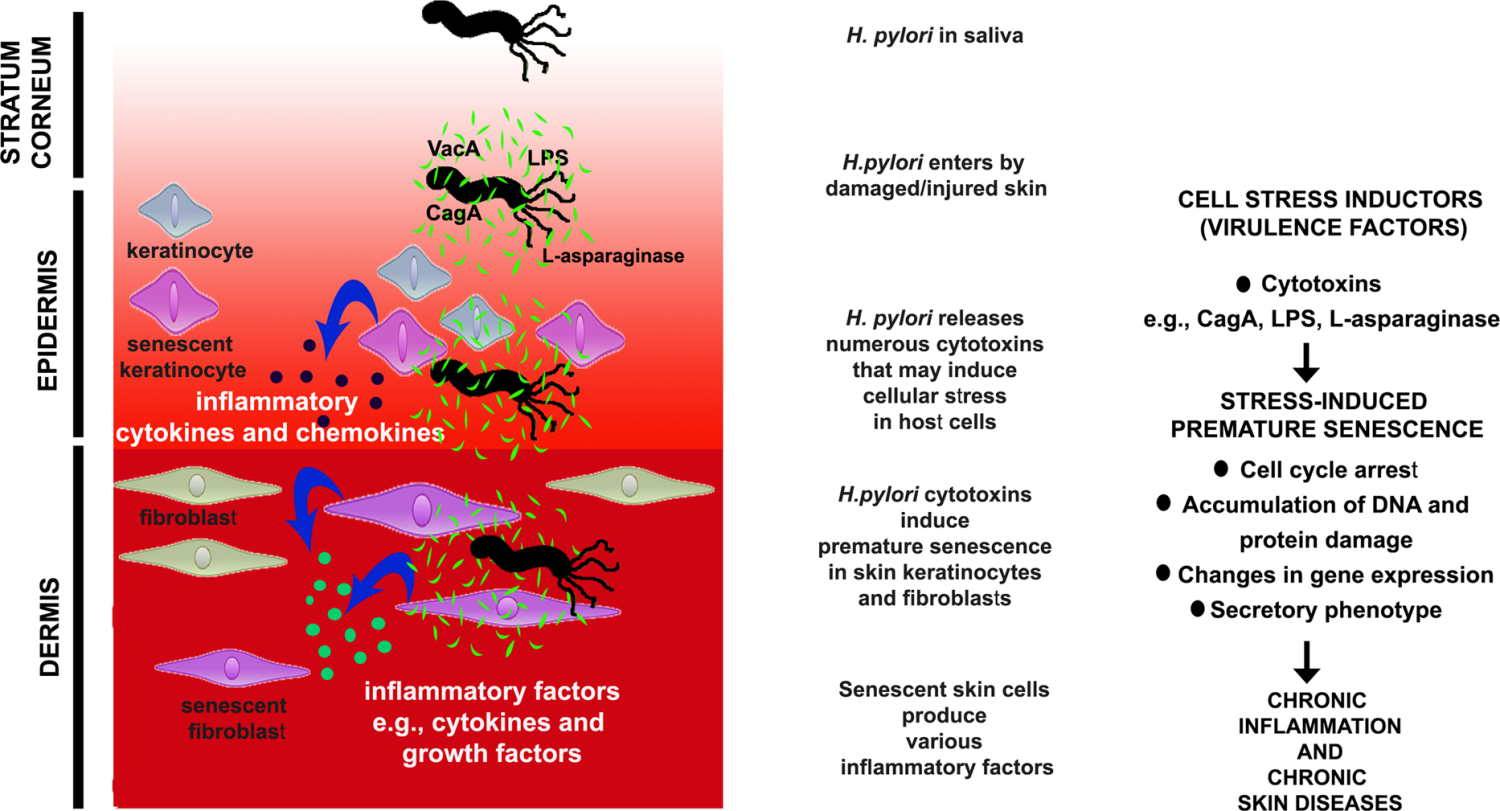
Molecular details of proposed hypothesis on *H. pylori*-mediated stress-induced premature senescence in skin cells and chronic skin diseases. Future studies are needed to verify the involvement of *H. pylori* and molecular players during SIPS-based chronic skin diseases

We have already mentioned that *H. pylori* cytotoxin CagA has been reported to stimulate cellular senescence in nonpolarized gastric epithelial cells (Saito et al. 2010). *H. pylori* l-asparaginase also inhibited the cell cycle of normal human diploid fibroblasts (Scotti et al. 2010). Of course, the question if such cell cycle arrest would be permanent and lead to cellular senescence needs to be addressed. In our opinion, gastrointestinal disorders and chronic skin diseases may have a common molecular basis that may be mediated by stress-induced premature senescence. We propose a hypothesis that *H. pylori* may promote stress-induced premature senescence in skin cells that in turn may lead to chronic inflammation and chronic skin diseases (Fig. 1).

Although, there are no direct evidences that *H. pylori* may induce SIPS in skin cells, one can speculate that such scenario is possible. First of all, *H. pylori* has been found in different human tissues including skin (Missall et al. 2012; Testerman and Morris 2014), as well as in saliva and faeces (Brown 2000). Moreover, *H. pylori* is able to tolerate a broad range of oxygen concentrations (Bury-Mone et al. 2006) and *H. pylori* possesses a plethora of enzyme activities that enables for survival at low pH in the stomach that may be also important during *H. pylori*-based skin infection, e.g. urease that converts urea to ammonium and carbon dioxide leading to local alkalization of acid pH in the stomach (Bury-Mone et al. 2001; Cornally et al. 2008; Tuzun et al. 2010). Thus, *H. pylori* is able to survive outside the gastrointestinal tract and its presence in other human tissues may affect host physiology and potentially provoke extragastric disorders. Moreover, the presence of *H. pylori* may promote redox imbalance (increased production of reactive oxygen species and reactive nitrogen species) (Handa et al. 2010) and DNA damage (Hanada et al. 2014; Koeppel et al. 2015; Toller et al. 2011), inflammation and epigenetic changes (Valenzuela et al. 2015) in host cells, all of which are triggers and/or biomarkers of cellular senescence.

In conclusion, it is postulated that the presence of *H. pylori* in the stomach may also affect other human tissues including skin and promote indirectly pathophysiological conditions outside the gastrointestinal tract (Magen and Delgado 2014; Testerman and Morris 2014). Therefore, more studies are still needed to verify our current knowledge on *H*. *pylori* as a systemic infectious factor and human skin cell responses to the presence of *H. pylori* as a part of complex host-pathogen interactions, especially *H*. *pylori*-induced premature senescence in skin cells, chronic inflammation and chronic skin diseases. Future studies might involve skin cell line models as well as clinical specimens and co-culture approach using intact *H. pylori* cells and isolated cytotoxins. Several biomarkers of cellular senescence could be then analyzed (Fig. 1). The presence of *H. pylori* in clinical skin samples could be also studied in an association with some biomarkers of cellular senescence in vivo.
